# Dignity Therapy Helps Terminally Ill Patients Maintain a Sense of Peace: Early Results of a Randomized Controlled Trial

**DOI:** 10.3389/fpsyg.2020.01468

**Published:** 2020-06-25

**Authors:** Luca Iani, Francesco De Vincenzo, Alice Maruelli, Harvey Max Chochinov, Matilde Ragghianti, Sieva Durante, Luigi Lombardo

**Affiliations:** ^1^Department of Human Sciences, European University of Rome, Rome, Italy; ^2^Psychology Unit, LILT and Center for Oncological Rehabilitation-CERION of Florence, Florence, Italy; ^3^Department of Psychiatry, University of Manitoba, Winnipeg, MB, Canada; ^4^U.O. di Cure Palliative, Fondazione Sanità e Ricerca, Rome, Italy

**Keywords:** dignity therapy, randomized controlled trial, spiritual well-being, demoralization, dignity-related distress, palliative care, end of life, terminal illness

## Abstract

**Introduction:** Dignity Therapy (DT) is a brief, individualized, narrative psychotherapy developed to reduce psychosocial and existential distress, and promote dignity, meaning, and hope in end of life patients. Previous studies have shown that DT was effective in reducing anxiety and depression, and improving dignity-related distress. However, less is known about its efficacy on spiritual well-being. The aim of this study is to contribute to the existing literature by investigating the effects of DT on specific dimensions of spiritual well-being, demoralization and dignity-related distress in a sample of terminally ill patients.

**Methods:** A randomized, controlled trial was conducted with 64 terminally ill patients who were randomly assigned to the intervention group (DT + standard palliative care) or the control group (standard palliative care alone). The primary outcome measures were Meaning, Peace, and Faith whereas the secondary outcome measures were (loss of) Meaning and purpose, Distress and coping ability, Existential distress, Psychological distress, and Physical distress. All measures were assessed at baseline (before the intervention), 7–10 and 15–20 days after the baseline assessment. The trial was registered with ClinicalTrials.gov (Protocol Record NCT04256239).

**Results:** The MANOVA yielded a significant effect for the Group X Time interaction. ANOVA with repeated measures showed a significant effect of time on peace and a significant Group X Time interaction effect on peace. *Post hoc* comparisons revealed that, while there was a decrease in peace from pre-treatment to follow-up and from post-treatment to follow-up in the control group, there was no such trend in the intervention group.

**Discussion:** This study provides initial evidence that patients in the DT intervention maintained similar levels of peace from pre-test to follow-up, whereas patients in the control group showed a decrease in peace during the same time period. We did not find significant longitudinal changes in measures of meaning, faith, loss of meaning and purpose, distress and coping ability, existential, psychological and physical distress. The findings of our study are of relevance in palliative care and suggest the potential clinical utility of DT, since they offer evidence for the importance of this intervention in maintaining peace of mind for terminally ill patients.

## Introduction

Over 19 million adults are in need of palliative care at the end of life worldwide, and higher distribution rates were observed in European and Western Pacific regions (Connor and Sepulveda, 2014). Palliative care is the holistic approach aimed at relieving suffering and improving quality of life in these patients and in their families. In this definition, the World Health Organization advocates that palliative care should meet the psychological and spiritual needs of patients^1^.

End of life issues among patients with life-threatening illnesses are receiving increasing attention within the literature. These patients have to face different challenges and frequently experience emotional and existential distress. Suffering at the end of life involves a complex and subjective experience, in which the understanding of physical, psychological, social, and spiritual needs is essential to inform meaningful and effective care (Krikorian et al., 2012; Bandeali et al., 2019). To mitigate suffering and to enhance end of life experience, health care professionals should assist patients in finding resources to fulfill these needs by providing a space for open discussion of concerns about death (Saracino et al., 2019).

Several interventions have been developed to address psychological and spiritual needs of terminally ill patients (LeMay and Wilson, 2008; Chen et al., 2018). Among them, Meaning Centered Psychotherapy, Life Review, and Dignity Therapy (DT) are three well-established, empirically validated interventions explicitly addressing existential concerns in life threatening illness (Saracino et al., 2019). DT is a brief, individualized, narrative psychotherapy which is based on an empirical model of dignity developed for end of life patients (Chochinov et al., 2002, 2005). This model provides a framework for understanding how terminally ill patients experience a sense of dignity and for developing interventions aimed at enhancing dignity in patients nearing death. It includes three categories, each of which contains different themes and sub-themes, that refer to experiences, feelings or events: (1) illness-related concerns (i.e., level of independence and symptom distress), (2) a dignity-conserving repertoire, which includes both dignity conserving perspectives (e.g., acceptance) and dignity conserving practices (e.g., living “in the moment”) and (3) a social dignity inventory, which refers to social concerns (e.g., burden to others). DT was designed to promote dignity, meaning and hope, and reduce psychological and existential distress. In DT, terminally ill patients are invited to discuss experiences of their life they would most want remembered or that matter most to them (Saracino et al., 2019).

Different systematic reviews and meta-analyses on DT showed promising but inconclusive results. Whereas terminally ill patients report benefits from DT for themselves and their families, the effects on psychological outcomes are less clear (Fitchett et al., 2015; Donato et al., 2016). For instance, a meta-analysis showed that DT did not reduce depression and anxiety of patients compared with the standard care (Xiao et al., 2019), whereas other reviews revealed a significant improvement of these psychological symptoms (Li et al., 2020) or mixed results (Sposato, 2016). The discrepancy between reported benefits and inconclusive evidence of DT may be due to several issues, such as low levels of distress at baseline (Martínez et al., 2017), the possibility of delayed effects (Fitchett et al., 2015), measures not sensitive enough to detect a psychological change (Sposato, 2016; Xiao et al., 2019) and lack of high quality study designs (Li et al., 2020). Overall, further clinical studies are needed along with the use of outcomes focusing on spiritual and existential domains (Fitchett et al., 2015; Donato et al., 2016). In this respect, the literature on end of life reveals the importance of demoralization, spiritual well-being, and dignity-related distress.

Spiritual well-being plays a pivotal role in the end of life experience as it may help patients to facilitate the search for meaning and to address questions about mortality (Ando et al., 2010). Conversely, unmet spiritual needs may cause depression as well as low sense of meaning and peace (Pearce et al., 2012). Spiritual well-being is defined as “a sense of meaning in life, harmony, peacefulness, and a sense of drawing strength and comfort from one’s faith” (Canada et al., 2008, p. 909). Several studies found that lower spiritual well-being was associated with hopelessness and depression in patients with life-threatening illnesses (Rodin et al., 2009; Gheihman et al., 2016). Studies investigating the effect of DT on spiritual well-being found no significant results (Sposato, 2016). For example, Chochinov et al. (2011) found no differences among Dignity Therapy (DT), Client Centered Care or Standard Palliative Care (SPC) on spiritual well-being after the intervention. It is worth noting, however, that Fitchett et al. (2015) conceptualized DT as an intervention with a strong spiritual component and suggested that its mechanisms may be associated with spiritual aspects of peoples’ lives. Moreover, patients’ discussions on dignity may include “matters of spiritual investment,” meaning and purpose (Chochinov and Cann, 2005).

Demoralization includes a variety of mental states, ranging from loss of confidence, to a mild loss of hope and purpose, to a state of despair, to severe demoralization (i.e., meaning and purpose are lost; Robinson et al., 2015). This loss of morale is the expression of existential challenges at the end of life and it is defined as “a morbid state of mind characterized by a considerable loss of meaning, hope, and purpose” (Kissane, 2014, p. 256). The demoralization syndrome is a common condition in patients with cancer or progressive diseases (Robinson et al., 2015) and is particularly relevant in terminally ill patients (Julião et al., 2016). There is strong evidence of associations between demoralization and depression, anxiety, physical symptoms, low quality of life, and desire for hastened death (Robinson et al., 2015; Julião et al., 2016). Demoralization was also associated with dignity-related distress and lower spiritual well-being (Bovero et al., 2019). It is worth noting, however, that demoralization is treatable (Griffith and Gaby, 2005) with a variety of interventions, including meaning-centered therapy and DT (Kissane, 2014). One RCT showed that DT decreased demoralization syndrome from baseline to post-intervention compared to standard palliative care (Julião et al., 2017).

Dignity refers to “the quality of being worthy of honor and respect” (Julião et al., 2017, p. 629). The extent to which physical, psychosocial, spiritual, and existential issues undermine patients’ dignity is defined as dignity-related distress (Chochinov et al., 2008), which may impact desire for hastened death (LeMay and Wilson, 2008). Studies showed that about one fifth of end of life cancer patients considered existential distress as a problem or major problem for the maintenance of their own dignity (Bovero et al., 2018). Moreover, patients with different advanced non-malignant disease had similar ratings of moderate to extreme loss of dignity (Chochinov et al., 2016). Research on the efficacy of DT for dignity-related distress produced mixed results (Donato et al., 2016; Li et al., 2020). For example, previous studies found no significant differences between DT and palliative care on dignity (e.g., Chochinov et al., 2011; Hall et al., 2011; Vuksanovic et al., 2017). Instead, Julião et al. (2017) found that patients who received DT showed a reduction in most of the Patient Dignity Inventory (PDI) items compared with patients in the control group.

In summary, there is no sufficient and consistent evidence for whether DT would influence spiritual well-being, demoralization and loss of dignity in terminally ill patients. The present study aims to contribute to the existing literature by investigating the efficacy of DT for specific dimensions of spiritual well-being, demoralization and dignity-related distress in a sample of terminally ill patients. Our first hypothesis was that only DT is associated with improvements, or at least stabilization in spiritual well-being dimensions scores that showed progressive worsening in controls. The second hypothesis was that DT yields the same pattern of results for demoralization and dignity-related distress as those expected for spiritual well-being.

## Materials and Methods

### Design and Sample

We conducted an RCT with terminally ill patients assigned to DT plus SPC or SPC alone. Patients were recruited between February 2018 and May 2020 from the Palliative Care Unit of Fondazione Sanità e Ricerca, Rome, and were mainly referred from hospitals, long-term care, nursing homes, and rest homes. Ethical approval was obtained from the local ethical committee (N. 0085021/2018). The trial was registered with ClinicalTrials.gov (Protocol Record NCT04256239) and was designed in accordance with the Consolidated Standards of Reporting Trials (CONSORT). All patients gave informed consent for the study in accordance with the Declaration of Helsinki. Eligibility criteria were age over 18, diagnosis of life-threatening disease with a prognosis ranging from 1 to 6 months (based on the evaluation of physicians who referred patients), no evidence of dementia (as determined by retrospective assessments), the ability to read and speak Italian and provide written informed consent, and the availability for six to seven research encounters over the period of 3 weeks. Exclusion criteria were fatigue, psychotic illness, dementia, severe neurological impairment, and a Karnofsky Performance Status (KPS) lower than 30. The presence of fatigue and KPS were based on clinician judgment, whereas the assessment of the other exclusion criteria was based on discharge medical records obtained from previous hospitalizations at other facilities. The patient’s awareness of diagnosis and prognosis was assessed through clinical interviews with the patient and interviews with family caregivers.

A recent meta-analysis showed that existential therapies had an average effect size of 0.65 on meaning in life (Vos et al., 2015). Power analysis showed that with an alpha of 0.017, based on Bonferroni correction to the significance level (accounting for having three primary outcomes), and a power of 0.80, we needed a sample of 86 participants to detect an effect size of 0.65 and higher.

A researcher not involved in the recruitment process (first author) assigned participants to the intervention group (DT plus SPC) or to the control group (SPC alone) using computer-generated random numbers. The first author had no information about the care recipient at the time of randomization.

A trained research assistant (S.D.) conducted assessments at baseline, 7–10 and 15–20 days after the baseline assessment. The blinding of outcome assessor to the randomization allocation was not possible in this study.

### Interventions

#### Dignity Therapy

DT is a short-term psychotherapy aimed at improving patients’ sense of personhood, purpose, meaning, and self-worth and reducing psychosocial and existential distress (Chochinov et al., 2005). DT is based on an interview guided by 10 questions that provide patients with the opportunity to address aspects of life they feel most meaningful or proudest of and to speak about things they need to say or they would most want remembered. Therapy sessions, lasting between 20 and 60 min, were offered at the patients’ bedside and audiotaped, and were conducted by a trained psycho-oncologist experienced in working with end of life patients (L.L.). The therapist was trained in DT techniques by a supervisor (A.M.), who previously attended training workshops conducted by H. Chochinov, using a standardized procedure. The supervisor, who was experienced in providing DT supervision, also reviewed protocol adherence and fidelity. After each therapy session, the audiotaped interview data were transcribed verbatim by a different psychologist (M.R.) and edited and reshaped into a written narrative by an expert in DT (A.M.) over the course of the next 2–3 days. Once the editing process was completed, another session was held to allow the therapist to read the “generativity document” to the patient and to make any editorial changes he/she deemed necessary. The final version of the generativity document was given to the patient to bequeath it to individuals of their choosing (e.g., friends, family members).

#### Standard Palliative Care

Standard palliative care was performed by a multidisciplinary care team composed of a palliative doctor, a psycho-oncologist, a nurse, a physiotherapist, a healthcare assistant, a social assistant, a volunteer and a spiritual assistant, tailoring care to the needs of patients and their families. In particular, the palliative care team provided comprehensive care for all patients throughout their hospital stay, including regular assessment and management of emotional and physical symptoms, clinical interviews, and psychological support for patients and their family.

### Measures

#### Primary Outcomes

The Functional Assessment of Chronic Illness Therapy-Spiritual Well-Being Scale (FACIT-Sp: Canada et al., 2008; Rabitti et al., 2020) was used to assess meaning (i.e., purpose in life), peace (i.e., sense of harmony and peacefulness), and faith (i.e., sense of comfort and strength from one’s faith and spiritual beliefs; Peterman et al., 2002). Items are rated on a Likert-type scale ranging from 0 (*not at all*) to 4 (*very much*). Higher scores indicate greater spiritual well-being.

#### Secondary Outcomes

Demoralization was assessed with the Demoralization Scale-II (DS-II; Robinson et al., 2016) measuring Meaning and purpose and Distress and coping ability. The Meaning and purpose subscale measures the loss of purpose, role, and sense of worth of life, whereas the Distress and coping ability subscale measures general dysphoria, a mild loss of confidence, and a sense of incompetence. Items are rated on a three-point Likert scale (0 = *never*; 1 = *sometimes*; 2 = *often*). Higher scores indicate higher levels of demoralization. Dignity-related distress was assessed with the Patient Dignity Inventory (Chochinov et al., 2008; Grassi et al., 2017) measuring existential distress, psychological distress, and physical distress. Existential distress measures existential suffering and loss of meaning (e.g., sense of burden to others). Psychological distress measures psychological symptoms including depression, uncertainty about the future, anxiety, and lack of coping skills. Physical distress measures physical symptoms (e.g., pain, nausea). Items are rated on a 5-point scale, ranging from 1 (*not a problem*) to 5 (*an overwhelming problem*). Higher scores indicate higher levels of distress.

### Data Analysis

We used *t*-test and chi-square analyses to determine whether the groups were equivalent at baseline on demographic, clinical, and outcomes variables. We also computed *t*-test analysis to examine whether completers and non-completers were equivalent at baseline on outcomes variables. We conducted a 2 (group) X 3 (time [pre-treatment vs. post-treatment vs. follow-up]) repeated measures multivariate analysis of variance (MANOVA) for a set of variables (i.e., meaning, peace, faith, meaning and purpose, distress and coping ability, existential distress, psychological distress, physical distress). Significant main or interaction effects were followed by one-way ANOVAs and by *post hoc* comparisons using Bonferroni adjusted *t*-test. Occasional missing values were imputed by calculating, for each participant, the mean score of the subscale and then replaced.

## Results

**FIGURE 1:**
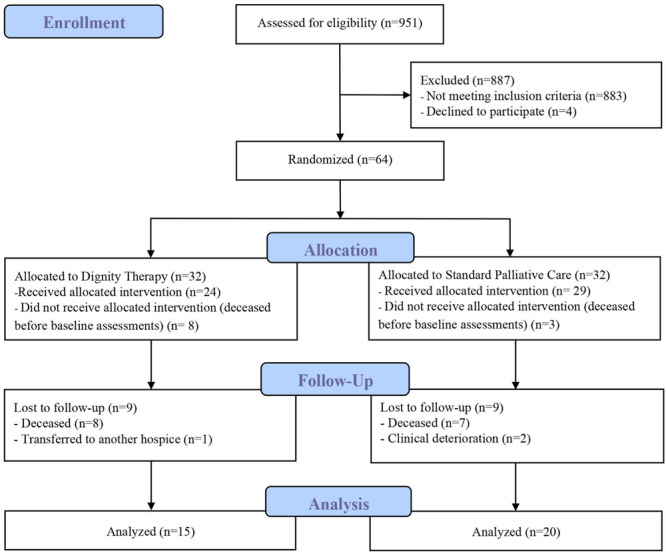
Flowchart diagram.

Nine-hundred fifty-one patients were assessed for eligibility. Of those, 883 did not meet inclusion criteria and 4 declined to participate because they were not interested in the study. A total of 64 patients were randomized; following randomization, 11 dropped out before baseline assessment (deceased) and 18 after post-treatment assessment (15 deceased, 1 transferred to another hospice and 2 withdrawn because of clinical deterioration). Thus, 35 patients completed the study (see Figure 1).

### Preliminary Analyses

Table 1 provides descriptive statistics on demographic and clinical characteristics of the 35 participants who completed the program. The total sample consisted of 21 women and 14 men with a mean age of 75.1 years (*SD* = 10.7). The majority of participants were unmarried, separated, or widowed (65.7%), had a high education level (31.4%), and had a Karnofsky Performance Status of 30 (42.9%). There were no significant differences between intervention and control groups on any variable. Moreover, participants who completed the study and those who dropped out did not differ on any variable except for Meaning and Purpose, which was higher in completers (*M* = 5.1, *SD* = 3.7) as compared to non-completers (*M* = 2.9, *SD* = 3.3, *t* [37.6] = -2.19, *p* = 0.035).

**TABLE 1:** Demographic and clinical variables according to treatment condition at baseline.

Variable, *n* (%)	Intervention group	Control group	Total sample	*t* or *X*^2^	*p*
	(*n* = 15)	(*n* = 20)	(*n* = 35)		
Age, *M* (*SD*)	74.6 (13.4)	75.4 (8.6)	75.1 (10.7)	*t*(22.4) = 0.21	0.832
Gender				*X*^2^(1, *N* = 35) = 0.00	1.000
Male	6 (40.0)	8 (40.0)	14 (40.0)		
Female	9 (60.0)	12 (60.0)	21 (60.0)		
Marital status				*X*^2^(1, *N* = 35) = 0.68	0.411
Unmarried/Widowed/Separated	11 (73.3)	12 (60.0)	23 (65.7)		
Married	4 (26.7)	8 (40.0)	12 (34.3)		
Education level				*X*^2^(4, *N* = 35) = 4.29	0.368
No title	1 (6.7)	4 (20.0)	5 (14.3)		
Primary school level	3 (20.0)	3 (15.0)	6 (17.1)		
Middle school level	4 (26.7)	1 (5.0)	5 (14.3)		
High school level	4 (26.7)	7 (35.0)	11 (31.4)		
Master degree	3 (20.0)	5 (25.0)	8 (22.9)		
Disease				*X*^2^(12, *N* = 35) = 8.13	0.775
Cancer-unknown origin	1 (6.7)	0 (0.0)	1 (2.9)		
Cancer-lung	2 (13.3)	6 (30.0)	8 (22.9)		
Cancer-pancreas	3 (20.0)	2 (10.0)	5 (14.3)		
Cancer-gallbladder	1 (6.7)	2 (10.0)	3 (8.6)		
Cancer-colon	1 (6.7)	1 (5.0)	2 (5.7)		
Cancer-breast	3 (20.0)	4 (20.0)	7 (20.0)		
Cancer-bladder	1 (6.7)	1 (5.0)	2 (5.7)		
Cancer-peritoneum	1 (6.7)	0 (0.0)	1 (2.9)		
Cancer-ovary	0 (0.0)	1 (5.0)	1 (2.9)		
Cancer-uterus	1 (6.7)	0 (0.0)	1 (2.9)		
Cancer-brain	0 (0.0)	1 (5.0)	1 (2.9)		
Cancer-liver	0 (0.0)	1 (5.0)	1 (2.9)		
Amiotrophic lateral sclerosis	1 (6.7)	1 (5.0)	2 (5.7)		
Karnowsky performance status, *M* (*SD*)	38.0 (7.7)	38.0 (10.0)	38.0 (9.0)	*t*(33.0) = 0.00	1.000
Time since first diagnosis (months), *M* (*SD*)	24.4 (23.5)	15.1 (15.3)	19.1 (19.5)	*t*(22.6) = -1.33	0.196

### Efficacy of Dignity Therapy

**FIGURE 2:**
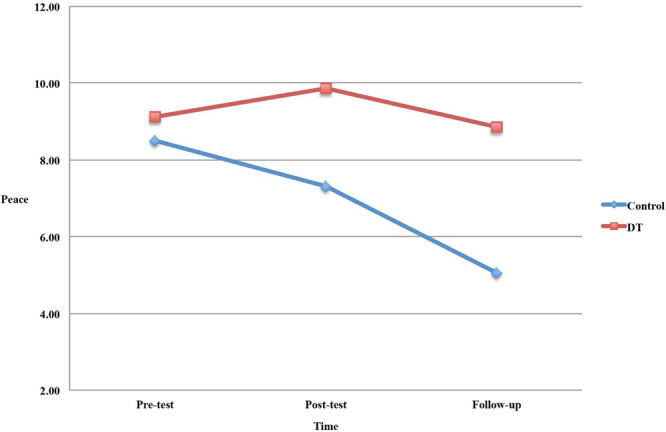
Effect of DT on sense of peace.

Table 2 presents the means and standard deviations of the dependent variables across time points. The MANOVA yielded a significant effect for the Group X Time interaction, *F*(16,118) = 2.34, *p* = 0.005, $\eta_{\text{p}}^{2}$ = 0.24. ANOVA with repeated measures showed a significant effect of time on peace, *F*(2,66) = 7.82, *p* = 0.001, $\eta_{\text{p}}^{2}$ = 0.19, and a significant Group X Time interaction effect on peace, *F*(2,66) = 4.91, *p* = 0.010, $\eta_{\text{p}}^{2}$ = 0.13. *Post hoc* comparisons revealed that, while there was a decrease in peace from pre-treatment to follow-up (*p* < 0.001) and from post-treatment to follow-up (*p* = 0.006) in the control group, there was no such trend in the intervention group. ANOVA with repeated measures also showed a significant Group X Time interaction effect on existential distress, *F*(2,66) = 4.15, *p* = 0.020, $\eta_{\text{p}}^{2}$ = 0.11. *Post hoc* comparisons revealed that there were no differences among time points on existential distress for both groups. Figure 2 shows group changes over time on peace.

**TABLE 2:** Means and standard deviations (in parentheses) for outcome measures in dignity therapy and control groups at the three assessment times.

Outcome measures	Dignity therapy (*n* = 14)			Control group (*n* = 15)		
	Pre-test	Post-test	Follow-up	Pre-test	Post-test	Follow-up
Meaning	13.3 (4.8)	13.7 (3.8)	12.3 (3.8)	11.4 (3.0)	10.7 (3.6)	10.0 (3.4)
Peace	9.1 (3.7)	9.9 (3.1)	8.9 (2.8)	8.5 (3.5)	7.3 (3.1)	5.0 (2.7)
Faith	4.8 (4.2)	5.9 (3.7)	4.7 (3.2)	5.0 (3.7)	4.5 (3.5)	4.1 (3.0)
Meaning and purpose	4.1 (3.1)	3.8 (4.5)	4.5 (4.2)	5.9 (3.9)	6.2 (4.4)	7.2 (4.3)
Distress and coping ability	5.7 (2.8)	3.9 (3.3)	5.7 (3.6)	6.4 (3.3)	6.8 (3.5)	6.5 (3.1)
Existential distress	24.1 (7.0)	20.7 (8.6)	22.7 (6.5)	22.8 (6.2)	25.2 (7.7)	22.7 (6.5)
Psychological distress	19.4 (6.5)	16.7 (7.1)	17.3 (5.9)	17.7 (5.0)	19.8 (6.4)	19.1 (6.6)
Physical distress	12.3 (6.2)	10.8 (4.9)	10.6 (5.2)	12.7 (4.9)	12.5 (4.1)	12.8 (4.5)

## Discussion

The aim of the present RCT was to examine the efficacy of DT for specific dimensions of spiritual well-being, demoralization and dignity-related distress in a sample of terminally ill patients. This study provides initial evidence that patients in the DT intervention maintained similar levels of peace from pre-test to follow-up, whereas patients in the control group showed a decrease in peace during the same time period. We did not find significant longitudinal changes in measures of meaning, faith, loss of meaning and purpose, distress and coping ability, existential, psychological and physical distress.

The stabilization and the progressive worsening of peace in DT and control groups, respectively, is a new finding. Previous studies reported that spiritual well-being (e.g., Chochinov et al., 2011) was not directly influenced by DT. Peace, or having a state of tranquility or serenity, is a kind of acceptance that involves a sense of reconciling with one’s adverse life circumstances (Whitford and Olver, 2012). Patients in the DT group seem to have concentrated their energy on remaining balanced and achieving a sense of peace and acceptance at the end of life. Thus, according to the Dignity Model, this finding suggests that DT may have helped patients maintain a continuity of self and “feel that they will have left something of value behind” (Saracino et al., 2019, p. 22). The worsening of peace in control group may be due to the fact that SPC itself did not specifically address sense of comfort, harmony or tranquility of terminally ill patients. Therefore, these patients may experience the awareness of imminent death as distressing, thereby reducing their sense of peace. Previous research suggested that the effect of therapies on the feeling of peacefulness has not been well-studied in palliative settings (Austin and MacLeod, 2017). The findings of this study suggest that examining spiritual well-being at the facet level (i.e., sense of meaning, peace, and faith) provides a better understanding of the extent to which each dimension is changeable after DT intervention.

DT intervention did not have a significant effect on meaning and faith compared to SPC. These results are consistent with previous research showing that spiritual well-being did not improve after DT (Sposato, 2016) and may reflect the possibility of delayed effects (Fitchett et al., 2015). Meaning is a stable characteristic (Steger and Kashdan, 2007; Davis and Novoa, 2013) that may require more commitment and time to change. Given that “faith is an integral part of one’s character or personality” (Fowler, 1981, p. 92), we hypothesize that also faith is not easily changeable through DT. These deeply rooted components of spiritual well-being may be malleable over time as patients reflect on the DT experience or share their legacy document with significant others (Fitchett et al., 2015).

There were no differences between DT and control groups on demoralization. This is an unexpected finding, as a previous study found that patients who received DT experienced significant reductions in demoralization syndrome compared to SPC patients (Julião et al., 2017). In our study, there was little room for improvement because demoralization was low at baseline whereas it was high in Julião et al.’s (2017) study. We hypothesize that demoralization scores were so low in our sample because when patients were admitted to the Palliative Care Unit, it has come as a great relief for them since they seemed to have high levels of hope to improve their clinical status. Moreover, in the Italian cultural context, patients’ relatives tend to provide high levels of comfort and support to terminally ill people and this may have buffered demoralization. Finally, patients who experience high levels of demoralization may be less likely to participate in DT (Fitchett et al., 2015). An alternative explanation of this finding is that these inconsistent results may be due to the different measurement instruments used. We measured this maladaptive coping response with the demoralization scale, which is considered a useful clinical and research tool when patient populations are at risk of demoralization (Robinson et al., 2016). Instead, Julião et al. (2017) measured demoralization syndrome by summing five diagnostic criteria (Kissane et al., 2001) and found that DT significantly decreased the prevalence of demoralization syndrome from baseline to post-intervention (Julião et al., 2017). As demoralization is frequently observed in terminally ill patients, more research is needed to verify whether DT interventions may reduce this poor coping response, characterized by hopelessness and helplessness, together with loss of meaning and purpose in life (Robinson et al., 2016).

The hypothesis that DT would be associated with improvements, or at least stabilization in dignity-related distress was not supported by the data. This finding is consistent with most previous research showing that there were no differences between DT and control groups on dignity-related distress (Chochinov et al., 2011; Hall et al., 2011; Vuksanovic et al., 2017). Our results suggest that dignity-related distress scores measured at baseline were relatively low, thus precluding DT from having any effect on such outcomes. Conversely, Julião et al. (2017) found that DT patients reported a reduction in most of the PDI items compared with participants in the control group. This discrepancy was likely due to the fact that participants in the latter study reported high dignity-related distress at baseline, although this choice may likely exacerbate issues with recruitment (Fitchett et al., 2015). More research is needed as to whether future DT studies targeting patients with elevated loss of dignity at baseline would have benefits that outweigh the costs related to recruitment.

This study has some limitations. First, the small sample size limited the power of the study. Second, there was a high attrition rate, mainly due to patients’ death. The results did not show any difference between dropouts and completers in baseline scores, with the exception of Meaning and purpose, completers having higher scores, which is likely due to chance. Third, the research assistant who conducted assessments was not blind to the treatment assignment and the possibility of rater bias should be considered. However, any assessment of terminally ill patients makes it difficult for evaluators to be “blind” to the treatment condition of the patient. Fourth, our sample consisted primarily of terminally ill cancer patients and results cannot be generalized to other patients. Notwithstanding these limitations, the present study provides initial evidence that peace may be susceptible to change through DT in the short term. The sense of inner peace is essential for patients’ end-of-life experience. As reported by Whitford and Olver (2012), “Only those that were suffering reported high levels of peace and were making the most of their lives, like the suffering was a wakeup call, a reminder not to take life for granted, a reminder to concentrate one’s energy on remaining balanced” (p. 609). The findings of our study are of relevance in palliative care and suggest the potential clinical utility of DT, since they offer evidence for the importance of this intervention in maintaining peace in terminally ill patients.

## Data Availability Statement

The datasets generated for this study are available on request to the corresponding author.

## Ethics Statement

The studies involving human participants were reviewed and approved by Comitato Etico Lazio 2. The patients/participants provided their written informed consent to participate in this study.

## Author Contributions

LI designed the study, analyzed the data, and wrote the manuscript. FD wrote the manuscript and collaborated in the editing of the final manuscript. AM designed the study and collaborated in the editing of the final manuscript. HC collaborated in the editing of the final manuscript. MR collaborated in the editing of the final manuscript. SD executed the study. LL designed and executed the study and collaborated in the editing of the final manuscript. All authors contributed to the article and approved the submitted version.

## Conflict of Interest

The authors declare that the research was conducted in the absence of any commercial or financial relationships that could be construed as a potential conflict of interest.

## Footnotes

1 World Health Organization (WHO). Definition of Palliative Care. Available at: https://www.who.int/cancer/palliative/definition/en/ [Accessed February 4, 2020].
